# Comparative assessment of methods for metagenomic DNA isolation from soils of different crop growing fields

**DOI:** 10.1007/s13205-016-0543-2

**Published:** 2016-10-12

**Authors:** Aiman Tanveer, Sangeeta Yadav, Dinesh Yadav

**Affiliations:** Department of Biotechnology, D.D.U Gorakhpur University, Gorakhpur, UP 273 009 India

**Keywords:** Metagenomics, Genomic DNA, Soil, Quantitative, Qualitative

## Abstract

The isolation of good quality metagenomic DNA from diverse soil, in appreciable amount, is a prerequisite for metagenomics. The availability of commercial kits for isolation of genomic DNAs from soil has drastically expedited the application of metagenomics approach for identifying novel sources of industrially important enzymes. The quantitative and qualitative assessment of metagenomic DNA isolated using either the manual method or the kit-based method should be performed prior to its use in downstream applications. The metagenomic DNA isolated from six different soil samples, using three methods, were analyzed in terms of yield, quality and downstream application as template for PCR amplification. The yield of DNA was approximately 3.52, 7.35, and 232.42 μg of DNA per gram of soil sample for the kit-based method, kit-modified method, and manual method, respectively. The manual method seems to be promising based on better yield and lesser humic acid content than the other two methods. The maximum yield was obtained in the soil collected from teak forest with all the three methods, indicating maximum microbial content and diversity. Furthermore, in terms of its suitability as template DNA for PCR amplification using 16S RNA primer, all methods are equally well. Thus, comparative assessment of three methods revealed suitability of manual method based on DNA yield and humic acid content, which could be important for many downstream applications like library preparations during metageomics approach.

## Introduction

Soil comprises of complex biological ecosystem containing wide plethora of microbial entities. The microbial population is known to be a vast reservoir of numerous biomolecules, biocatalysts, antibiotics, and antibiotic resistance genes (Daniel 2005; Riesenfeld et al. 2004; Simon and Daniel 2011). The immense biological treasure stored in the ecosystem either in soil, water, and air needs to be explored. Researchers have attempted to explore this rich ecosystem by culturing the microbial strains under laboratory conditions but this account for less 1 % of the total microbial diversity (Handelsman 2004). In order to assess the remaining microbial diversity, ‘metagenomics’ is the only alternative and has been extensively reviewed over the years (Fernandez-Arrojo et al. 2010; Schmeisser et al. 2007; Steele et al. 2009; Uchiyama and Miyazaki 2009). Metagenomic involves analysis of DNA from diverse soil, marine, and airborne habitats. DNA extracted from the microbial communities is cloned in the hosts which can be easily cultured in laboratory conditions (Handelsman 2004). The library is then screened for the desired biomolecules.

The isolation of metagenomic DNA from soil is an important consideration and needs standardization. Since soil is rich in organic matter like humic acid and other inhibitory substances, which hinder downstream applications like PCR amplification and cloning, several protocols have been reported in literature (Berthelet et al. 1996; Gutiérrez-Lucas et al. 2014; Nair et al. 2014; Picard et al. 1992).

The study is primarily focused on the assessment of methods for the isolation of metagenomic DNA from different soil samples, for identifying novel sources of industrially important enzymes of pectinases group, especially pectin lyases (Yadav et al. 2009). Using metagenomic approach, an attempt has been made to access the quantity and quality of soil genomic DNA isolated from different sources using different methods. In this study, a comparative assessment of genomic DNA isolated by the kit-based and manual methods from six different soil samples consisting of different crops is discussed.

## Materials and methods

### Soil samples

A total of six soil samples, five representing soils from agricultural field with mustard, broad bean, pea, sugarcane, and wheat crops, and one soil sample representing the teak dominated Kushmi forest located in Gorakhpur, Uttar Pradesh, India (Latitude 26°13′N and 27°29′N and Longitude 83°05′E and 83°56′E), were used in this study. The greater part of the district falls in the eastern sector of the Indo-Gangetic plain containing older alluvium (Bangar) and new alluvium (Khadar) soil types.

### Genomic DNA extraction

Metagenomic DNA was extracted by three different methods, namely, HiPurA soil DNA isolation kit (Himedia), modified HiPurA soil DNA isolation kit, and manual method (Nair et al. 2014) using soil samples from six different locations.

#### Method 1: HiPurA soil DNA isolation kit (Himedia)

The metagenomic DNA was extracted as per manufacturer’s instructions. Both mechanical actions, i.e., bead beating, heating and chemical lysis is involved in the process. The sample is subjected to cycle of intensive vortexing, heating and vortexing. The inhibitor removal solution is added for removing impurities like humic acid and other PCR inhibitors. The supernatant after addition of the binding solution was loaded on the spin column. The column was centrifuged to bind the DNA to spin column and the column was washed with wash buffer provided in the kit. The DNA is finally eluted with the elution buffer and could be stored at –20 °C freezer.

#### Method 2: Modified HiPurA soil DNA isolation kit (Himedia)

A slight modification of the protocol mentioned in HiPurA soil DNA isolation kit (Himedia), namely, addition of RNase A (1 μg/ml) along with the inhibitor removal solution and incubating it at 37 °C for 1 h. Second, along with the wash buffers provided in the kit, additional washes were performed with 70 % ethanol twice. The sample was eluted in the elution buffer preheated at 65 °C.

#### Method 3: Manual method

This method has been reported by Volossiouk et al. (1995) and Nair et al. (2014). In this method, the soil was ground to fine powder in aseptic conditions and suspended in 0.4 % w/v solution of skimmed milk. The solution was vortexed well and centrifuged at 12,000*g* for 10 min. To the supernatant, 2 ml of SDS extraction buffer (0.3 % SDS in 0.14 M NaCl, 50 mM sodium acetate, pH 5.1) was added and mixed well by vortexing. Equal volume of tris saturated phenol was added and vortexed again for 2 min. Tube was centrifuged at 12,000*g* for 10 min and supernatant was collected. DNA was precipitated with equal volume of ice-cold isopropanol at –20 °C for 1 h. The DNA pellet was recovered by centrifugation at 12,000*g* for 10 min. Pellet was washed twice with 70 % ethanol and air dried. Pellet was dissolves in sterile water and stored at –20 °C deep freezer.

### Quantitative and qualitative assessment of metagenomic DNA

The isolated metagenomic DNA was analyzed by standard agarose gel electrophoresis loading equal quantities of DNA on the agarose gel along with λ *Hin*dIII digest marker (Maniatis et al. 1982). Purity and concentration of DNA were estimated by Nanodrop. The yield of extracted DNA was determined by measuring absorbance at wavelength of 260 nm. The purity of corresponding DNA samples was determined by the calculating *A*_260_/*A*_280_ (DNA/protein) and *A*_260_/*A*_230_ (DNA/humic acid) ratios to determine protein and humic acid contamination, respectively. *A*_260_/*A*_280_ ratio of less than 1.8 indicates protein contamination (Maniatis et al. 1982), while *A*_260_/*A*_230_ value less than 2 indicates humic acid contamination (Ning et al. 2009). Furthermore, the quality of genomic DNA isolated from soil samples were assessed for PCR amplification using 16S rRNA gene specific primer. The primer sequences are, forward primer: AGAGTTTGATCCTGGCTCAG; reverse primer: TACGGYTACCGTGTTACGACTTK.

### Statistical analysis

The isolation of metagenomic DNA using different methods from different soil samples were repeated thrice and values of absorbance and the absorbance ratios according calculated for mean and standard deviations. Microsoft excel was used for all the statistical calculations and graph preparation.

## Results and discussion

The key step in the metagenomic workflow is the isolation of high-quality metagenomic DNA with good yield. This study was focused on the selection of optimal method for extraction of metagenomic DNAs from various crop growing fields of north-eastern Tarai Gorakhpur region of Uttar Pradesh, India. Isolation of good quality DNA for application in other downstream applications is a challenging task. One of the hurdles in isolation of high-quality DNA is the co-purification of other contaminating molecules present in the soil like humic acid, heavy metals, and some pigments. Among these, humic acid creates the major menace as it is present in all the soil types playing major reservoir of organic carbon in soil. Irrespective of the soil source, around 0.7–3.3 mg/ml of humic acid contaminant, may be present in any crude DNA preparation (Tebbe and Vahjen 1993). Major reason for its co-purification is its three-dimensional structure with functional reactive groups through which it binds during the purification process (Stevenson 1976). Moreover, it shares the same properties as the DNA molecule, making it more difficult to separate from the metagenomic DNA during the isolation steps.

There are several reports of metagenomic DNA isolation from different soil samples. Classical methods comprises of both mechanical and chemical means for shearing the cells. Mechanical shearing in the form of bead beating (Dong et al. 2006), or heating at 65 °C (Zhou et al. 1996) or subjecting the cells to alternate cycle of freeze and thaw conditions (Tsai and Olson 1991) has been attempted. Various buffer combinations containing detergents are used for lysis and subsequent purification step in genomic DNA isolation. However, currently, commercial DNA isolation kits are becoming popular for isolation of good quality metagenomic DNA in lesser time. Irrespective of the commercial sources, the kits basically work on the principle of adsorption and desorption of DNA on the silica column in the presence of chaotropic salts. The isolated metagenomic DNAis routinely used for estimation of functional diversity, taxonomic classification, and community structure studies (Jimenez et al. 2012; Jung et al. 2016; Uroz et al. 2013).

**Fig. 1:**
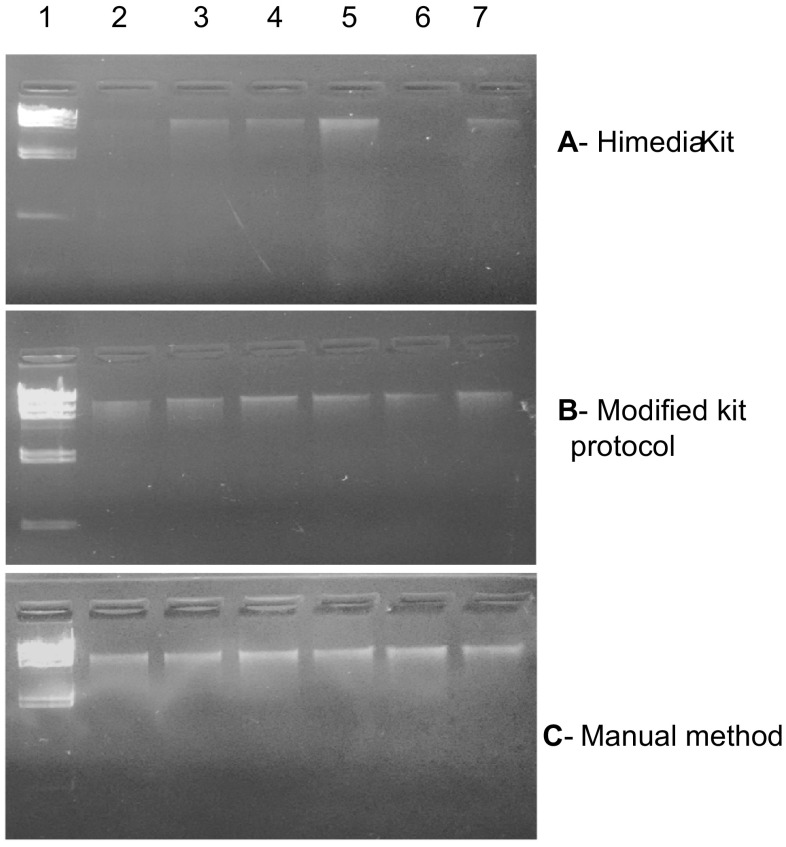
0.8 % agarose gel showing genomic DNA, isolated from different soil samples using, **a** Himedia kit, **b** modified kit protocol, and **c** manual method. *Lane 1* lambda-*Hin*dIII marker DNA, *lane 2* mustard field, *lane 3* teak forest, *lane 4* broad bean, *lane 5* pea field, *lane 6* sugarcane field, and *lane 7* wheat field

The metagenomic DNA isolated from six different soil samples, using three methods, were analyzed in terms of yield, quality and downstream application as template for PCR amplification. The yield of genomic DNA was comparatively higher for method 3, i.e., manual method (Nair et al. 2014) as compared to HiMedia kit-based method 1 and modified kit method 2 (Fig. 1). The selection of this manual method (Ogram et al. 1987) was based on reports of being the best method assessed during comparative analysis of five reported methods for soil DNA extraction (Nair et al. 2014).

The maximum yield was obtained in the soil collected from teak forest with all the three methods. This also indicates maximum microbial content and their diversity in the Teak forest. The quantity of DNA amounted to around 3.52, 7.35, and 232.42 μg of DNA per gram of soil sample for the kit-based method, kit-modified method, and manual method, respectively. The yield obtained by the manual method was much better than some other reported methods. Approximately 1.29 μg of DNA was extracted by the method reported by Zhou et al. (1996). Yeates et al. (1998) have used mechanical means for the isolation of the metagenomic DNA. They have obtained 3.42 and 1.47 μg of DNA per gram of soil using glass beads and sonicator, respectively. A very good yield of 746.46 μg of DNA per gram of soil has been reported by Tsai and Olson (1991).

**Fig. 2:**
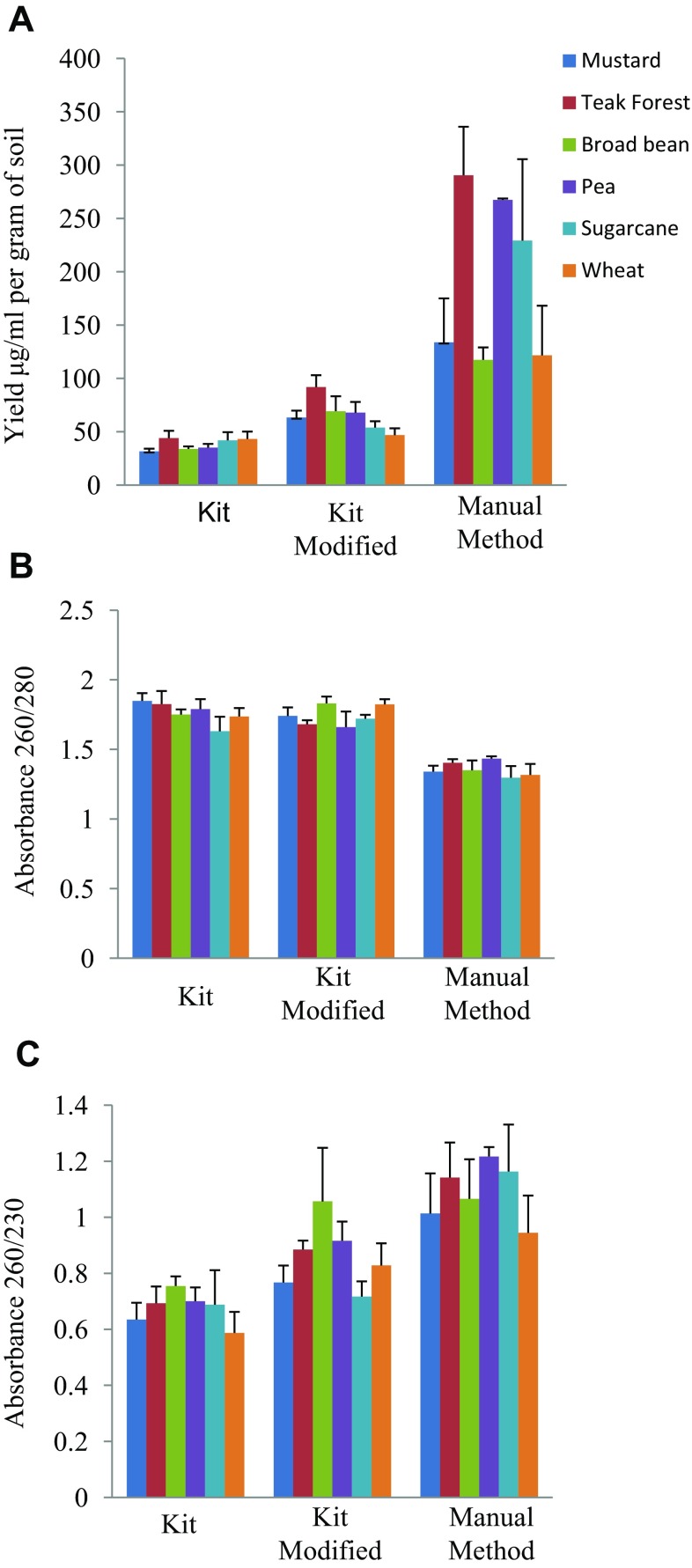
Comparative assessment of metagenomic DNA isolation methods for **a** yield, **b** protein contamination (Absorbance 260/280), and **c** humic acid contamination (Absorbance 260/230)

Irrespective of the methods used, the genomic DNA was of good quality with discrete bands, as visualized on agarose gel (Fig. 1). The qualities of DNA isolated by different methods from six different soil samples were further assessed for the presence of protein and humic acid contaminants. Manual method seems to be more efficient in the effective removal of humic acid contaminants (Fig. 2a) as compared to other methods tested, based on values of *A*_260_/*A*_230_ calculated for each samples. The *A*_260_/*A*_230_ ratio of less than two indicates the presence of humic acid contamination (Ning et al. 2009). For better performance of the kit-based methods, the humic acid and other possible contaminant in the isolated DNA can be minimized by additional washing steps by 70 % ethanol (Fig. 2c). Furthermore, the RNA co-purified by the purification procedure can be removed by RNase treatment. The protein contamination as analyzed by determining *A*_260_/*A*_280_ was also assessed for isolated metagenomic DNAs using different methods. The manual method (Method 3) seems to have more protein contamination than kit-based methods (Fig. 2b).

**Fig. 3:**
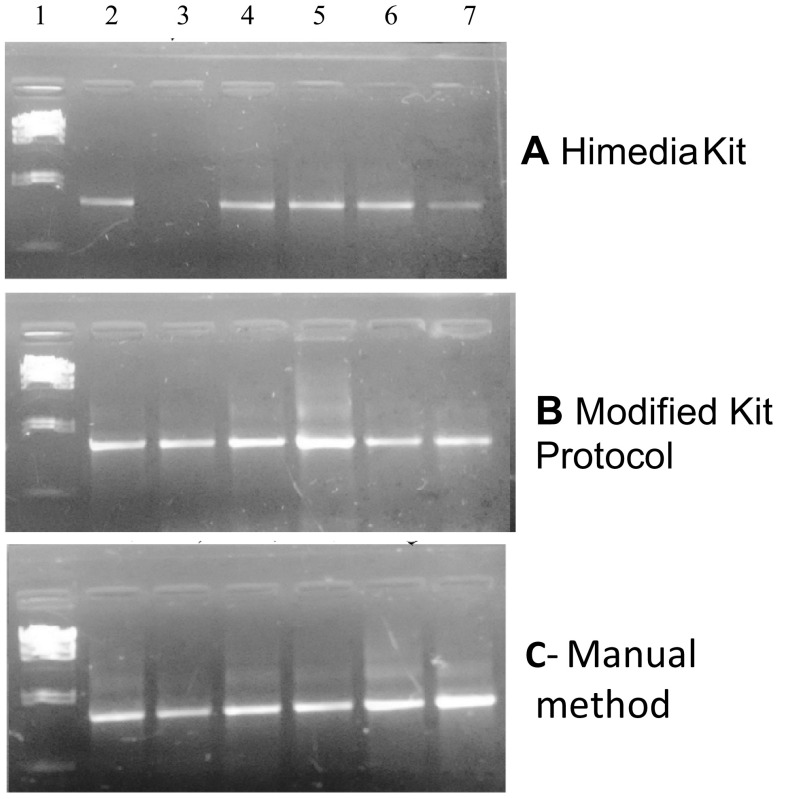
1.5 % agarose gel showing PCR amplification of 16S rRNA using genomic DNA isolated from different soil samples as template **a** Himedia kit, **b** modified kit protocol, and **c** manual method. *Lane 1* lambda-*Hin*dIII marker DNA, *lane 2* mustard field, *lane 3* teak forest, *lane 4* broad bean, *lane 5* pea field, *lane 6* sugarcane field, and *lane 7* wheat field

Quality of the isolated metagenomic DNAs was further analyzed for downstream application mainly as template for PCR amplification using 16S rRNA primer. In general, a high-quality DNA free from contaminants is a prerequisite for PCR amplification. Humic acid acts as an inhibitor in PCR by binding to the DNA molecule hindering the amplification of the DNA molecule (Opel et al. 2010); hence, the template soil DNA should be free of humic acid contamination. The PCR amplification using soil metagenomic DNAs with 16S rRNA primer resulted in expected size amplicons of 1.5-kb size (Fig. 3) irrespective of the isolation methods used, indicating the acceptability of all the methods tested.

Thus, in this study, it is quite evident that the manual method gives comparatively better yield along with lesser humic acid contaminants, as compared with the other two methods. Although all the methods tested are satisfactory as evident from the PCR amplification, the yield of metagenomic DNA is quite variable and this could be an important consideration for many downstream applications like library preparations during metageomics approach. Furthermore, these methods are equally suitable for different soil types used in this study.

Currently, there are no reports about the metagenomics studies of soil from the crop growing fields of Gorakhpur district of Uttar Pradesh, India. Since it is an agriculturally dominant region, metagenomic studies need to be carried out to look into the rich microbial diversity present in the soil. This is probably for the first time metagenomic studies have been performed from different soils of this region and quality checked by PCR amplification of 16S rRNA gene. The reproducibility of protocol for metagenomic DNA isolation is an important consideration and needs to properly investigated prior to applying metagenomic approaches for isolating novel sources of enzymes.
